# Making Big Sense From Big Data

**DOI:** 10.3389/fdata.2018.00005

**Published:** 2018-10-15

**Authors:** Thomas Hartung

**Affiliations:** Bloomberg School of Public Health, Johns Hopkins Baltimore, MD, United States

**Keywords:** medicine, public health, machine learning, artificial intelligence, learning methods

In 1980, in my last years of high school, I worked as a programmer in a small company for a bit of extra money. At the time, we received a first hard-disk, a Winchester model—$25,000 and as big as a fridge. Its capacity was 10 MB. Today, many of my photographs are larger. Yet in 1980, we wondered which small customer company would really need this…

I do not need to tell the audience interested in Big Data and Artificial Intelligence that times have changed. Today, it is sometimes cheaper to produce data than to store them. My own university, Johns Hopkins, is one of the leading whole patient genome sequencing centers. In condensed form a genome is about 100 GB—a lot considering that 100 million to 2 billion genomes sequenced by 2025 have been estimated^1^. Prices for generating sequences have dropped so incredibly (now to around $1,000 each) that Johns Hopkins is considering not to store the results of DNA sequencing after the end of studies but to keep the DNA to repeat the sequencing if needed again. Others are dealing with enormous data from high-content imaging, something particularly evident in radiology. Indeed, a recent prediction by the research group IDC estimates that the world will be creating 163 zettabytes of data a year by 2025^2^. Just to recall: a zettabyte is one trillion gigabytes. Yet as much as 30% of the entire world's stored data is generated in the health care industry and there is tremendous value behind such data: “*A single patient typically generates close to 80 megabytes each year in imaging and electronic medical record (EMR) data. This trove of data has obvious clinical, financial, and operational value for the health care industry, and the new value pathways that such data could enable have been estimated by McKinsey to be worth more than $300 billion annually in reduced costs alone”*^3^ The dawn of the “big data” era has clearly broken and its trends and dimensions are only accelerating.

I recently came across a witty quote from Dan Ariely, professor of psychology and behavioral economics at Duke University: “*Big Data is like teenage sex: everyone talks about it, nobody really knows how to do it, everyone thinks everyone else is doing it, so everyone claims they are doing it*”^4^ This will actually be one of the challenges we have to face—defining what big data and A.I. are and aren't, and how to do them properly. These challenges include identifying the biases in data and data extraction, the control of code, the documentation of data manipulation, the reporting of these processes and results, as well as the sharing of data and algorithms. Ultimately, we need to develop a culture of validation and this has to go beyond typical cross-validations splitting the dataset into a training and test set; truly independent datasets need to be identified to challenge our results and show their value beyond the dataset studied. This is not simple as we tend to incorporate available data into our datasets to increase statistical power and not leave them apart for such validation studies. Often it is therefore difficult to find such independent datasets of sufficient quality. There are other, much needed approaches like data-scrambling and sensitivity analyses, among others, to avoid over-fitting of data, but this is not yet a common standard in the field.

But the point is not about generating or storing Big Data—it's about squeezing sense out of them. It is about how to ensure the quality of data and the relevance of results. We need a culture of quality control and quality assurance, and of good practices, especially when having to trust machines to derive our results. Nate Silver, author and founder of the blog FiveThirtyEight, rightly pointed out that “*When human judgment and big data intersect there are some funny things that happen.”* And, in terms of technological developments, we are dealing with one of the fastest moving targets. The exchange of these advances across medical sectors needs to be fast. Indeed, most of these methods are agnostic of the type of data used. So, we can learn from each other even if we are interested in completely different aspects of medicine and public health. There are also ethical and moral dimensions. The German economist Klaus Schwab, founder and executive chairman of the World Economic Forum, warned us: “*We must address, individually and collectively, moral and ethical issues raised by cutting-edge research in artificial intelligence and biotechnology, which will enable significant life extension, designer babies, and memory extraction.”* In medicine and public health, wrong decisions from big data/A.I. can have devastating consequences. We just learned for example^5^ that IBM's Watson A.I. for cancer therapy recommended unsafe therapies. We have a moral obligation to deliver the right results and communicate them with their very real limitations to society and policy-makers. It is too easy to impress with big numbers and too difficult for non-experts to cross-check—big data need to adhere to clear standards of truthfulness and reliability.

The section on AI and Big Data in Medicine and Public Health, which is part of *Frontiers in Big Data* and *Frontiers in Artificial Intelligence*, wants to help exactly this. Our goal is the cross-fertilization between medical disciplines: to improve the information gain, not the data gain. An Open Access journal with fast turnover of articles and a high quality review seems ideally suited to serve this emerging community. Sure, the hype around these approaches allows publishing in many specialized journals, but there the communality is in the research areas not in the approach. The dedicated twin journals are focusing on the “communication needs” around Big Data and Big Computing. This will allow us to develop reporting standards and innovative ways of sharing access to data and its mining tools.

We also need to find ways to identify the gold nuggets, those reports where we get Big Sense from data mining. We need to trailblaze and find the best ways to build bridges and make approaches and/or results accessible and usable to others.

My aim is to establish a community-driven section, focused on AI and Big Data in Medicine and Public Health, and to provide an innovative platform of exchange of high-quality information—a trusted resource. We have made important first steps, especially by recruiting a fantastic team of associate editors and by piggybacking on the successful infrastructure of the Frontiers journal family. I am excited to see how the journal will shape itself and indeed the Big Data and A.I. community in biomedicine.

## Author contributions

The author confirms being the sole contributor of this work and has approved it for publication.

### Conflict of interest statement

The author declares that the research was conducted in the absence of any commercial or financial relationships that could be construed as a potential conflict of interest.

## Footnotes

^1^https://www.technologyreview.com/s/542806/how-do-genome-sequencing-centers-store-such-huge-amounts-of-data/.

^2^https://www.seagate.com/www-content/our-story/trends/files/Seagate-WP-DataAge2025-March-2017.pdf.

^3^https://catalyst.nejm.org/case-data-scientists-inside-health-care/.

^4^https://www.facebook.com/dan.ariely/posts/904383595868.

^5^https://www.statnews.com/2018/07/25/ibm-watson-recommended-unsafe-incorrect-treatments/.

